# Artificial Intelligence and Endobronchial Ultrasound for Lymph-Node Detection and Characterization: A Pioneer Multicenter Transatlantic Study

**DOI:** 10.3390/jcm15145356

**Published:** 2026-07-08

**Authors:** Miguel Mascarenhas, Gema Diaz Nuevo, Sara Lopes, Juan Puertas Sancho, Cristina Lopez Garcia-Gallo, Luiza Bicudo de Oliveira, Miriam Chinzon, Larissa Mercadante de Assis, Gustavo Ejima, Fauze Maluf-Filho, Matheus Ferreira Carvalho, Thomás Paiva Lima Dias, Isabele Alves Chirichela, Tiago Ribeiro, Francisco Mendes, Ana Pérez, Belén Agudo Castillo, António Pinto da Costa, Mariano González-Haba, João Fonseca, Guilherme Macedo, Eduardo Guimarães Hourneaux De Moura, João Pedro Ferreira, João Marcelo Lopes Toscano de Brito, Tiago Noguchi Machuca, Paulo Rogério Scordamaglio, Felipe Nominando Diniz Oliveira, Adelino F. Leite-Moreira

**Affiliations:** 1 Department of Community Medicine, Information and Health Decision Sciences, Faculty of Medicine, University of Porto, 4200-319 Porto, Portugal; 2 Precision Medicine Unit, Department of Gastroenterology, Unidade Local de Saúde de São João, 4200-319 Porto, Portugal; 3 World Gastroenterology Organization Gastroenterology and Hepatology Training Center, 4200-319 Porto, Portugal; 4 Department of Pneumology, Hospital Universitario Puerta de Hierro Majadahonda, 28222 Madrid, Spain; 5 Department of Thoracic Surgery, Instituto Português de Oncologia-Porto, 4200-072 Porto, Portugal; 6 Gastrointestinal Endoscopy Unit, Department of Gastroenterology, Hospital das Clínicas da Faculdade de Medicina da Universidade de São Paulo, São Paulo 05403-000, Brazilmiriam.chinzon@icloud.com (M.C.); ejimagustavo@gmail.com (G.E.); fauze.maluf@hc.fm.usp.br (F.M.-F.);; 7 Department of Thoracic Surgery, Hospital São Luiz-Rede D’Or, São Paulo 04544-000, Braziljoaomarcelo.ltb@hotmail.com (J.M.L.T.d.B.);; 8 Department of Medicine, Faculty of Medicine, University of Porto, 4200-319 Porto, Portugal; 9 Department of Digestive Endoscopy, Hospital Universitario Puerta de Hierro Majadahonda, 28222 Madrid, Spain; ana.perez.gs96@gmail.com (A.P.);; 10 Department of Mechanical Engineering, Faculty of Engineering, University of Porto, 4200-465 Porto, Portugal; 11 Department of Pneumology, Hospital São Luiz-Rede D’Or, São Paulo 04544-000, Brazil; 12 Department of Pneumology, Hospital das Clínicas da Faculdade de Medicina da Universidade de São Paulo, São Paulo 05403-900, Brazil; 13 Department of Surgery and Physiology, Faculty of Medicine, University of Porto, 4200-319 Porto, Portugal; 14 Cardiothoracic Surgery, Unidade Local de Saúde de São João, 4200-437 Porto, Portugal

**Keywords:** EBUS, bronchoscopy, lymph-nodes, artificial intelligence, lung cancer, deep learning, convolutional neural network

## Abstract

**Background/Objectives**: Accurate mediastinal and hilar lymph node (LN) assessment is central to lung cancer staging and treatment selection. Endobronchial ultrasound-guided transbronchial needle aspiration (EBUS-TBNA) has become the standard minimally invasive procedure for mediastinal staging, enabling real-time identification, characterization and sampling of LNs. Although effective, this technique remains operator dependent, and subject to interobserver variability. This study aims to report the development of a Vision Transformer-based AI model for automatic LN detection and classification on EBUS images. **Methods**: A multicenter, multidevice, retrospective study, using EBUS images of three referral centers (Spain and Brazil). The dataset included images of patients with benign and malignant LNs. Bounding-box annotations were applied for LN localization, and LNs were classified as benign or malignant according to histopathologic reference standards. Detection performance was evaluated with mean average precision, at an intersection over union threshold of 0.5 (mAP50). Characterization performance was assessed using accuracy, sensitivity, specificity, and area under the receiver operating characteristic curve (AUROC). **Results**: The dataset included 6492 images from 27 patients, of whom 16 had malignant LNs. In the test set, the model achieved a mAP50 of 0.79 for LN detection. For malignant characterization, the model achieved an accuracy of 90.6%, sensitivity of 85.4%, and an AUROC of 0.938. **Conclusions**: The results of this study support the feasibility of AI-assisted EBUS interpretation for nodal staging workflows and raise the possibility that AI-assisted EBUS could enhance diagnostic performance, while reducing operator variability. Larger prospective studies are needed before clinical implementation.

## 1. Introduction

Over the last years, bronchoscopy practice has expanded beyond conventional white-light inspection to include image-enhanced bronchoscopy, endobronchial ultrasound (EBUS), navigation systems, and advanced tissue acquisition techniques [1,2,3]. Diagnosis and management of a wide range of thoracic diseases, including lung cancer (LC), is, nowadays, highly dependent on pulmonary endoscopy. EBUS, particularly EBUS-transbronchial needle aspiration (EBUS-TBNA), is a primary diagnostic tool for mediastinal and hilar standing in LC patients. According to LC guidelines, EBUS must be performed in central tumors, tumors measuring 3 cm or greater, and when there are suspected lymph nodes (LNs): over 1 cm in computed tomography (CT), and/or positive in positron-emission tomography (PET) [3,4,5]. EBUS-TBNA has become a cornerstone for minimally invasive nodal staging in LC, providing minimally invasive access, with high sensitivity and specificity [3,6,7,8]. EBUS also informs us about resectability, oncologic planning, neoadjuvant treatment, perioperative regimens, and prognosis. However, regarding LN assessment, a more reproducible EBUS evaluation and report is warranted.

AI has shown strong performance in image recognition, lesion detection and characterization, reduction in interobserver variability and workflow optimization, across image-intensive specialties, including gastrointestinal endoscopy, radiology and pathology [3,9,10]. Machine learning (ML) and deep learning (DL) approaches are being increasingly used to support image interpretation, navigation and procedural decision-making in pulmonary endoscopy [3,9]. DL can automate detection, reduce subjectivity, and extract patterns beyond visual assessment. These capabilities are particularly relevant in procedures requiring real-time interpretation, such as EBUS: AI could help standardize node recognition, reduce subjectivity, and support sampling decisions in real time, with potential to improve mediastinal staging accuracy [9,10,11,12,13,14,15,16,17].

Recent evidence suggests that AI applications in pulmonary endoscopy encompass a broad spectrum of tasks, including computer-aided detection (CADe), computer-aided diagnosis (CADx), image enhancement, navigation assistance, quality assessment, procedural guidance, and prognostic prediction. However, despite the rapid growth of the field, the overall level of evidence remains limited by small datasets, retrospective study designs, heterogeneous methodologies, limited external validation, and the absence of prospective clinical implementation studies. These limitations currently represent major barriers to translation into routine clinical practice [3,9,10,11,12,13,14,15,16,17].

Existing AI work in bronchoscopy and thoracic imaging has focused mainly on radiology, navigation, or static image classification. In this way, compared with other endoscopic applications, AI for EBUS remains poorly developed with limited external validation and unresolved challenges related to workflow integration, and regulatory oversight [5,9,10,11,12,13,14,15,16,17]. Existing work is limited, often retrospective, single center, single device, with heterogeneous datasets, and focused only on classification rather than full CADe workflows [10,14,15,16,17].

Furthermore, recent reviews have highlighted that most published EBUS-AI systems have evaluated either LN characterization or image classification in isolation, whereas integrated frameworks capable of simultaneously performing LN localization and malignant characterization remain scarce. In addition, multicenter and multidevice validation studies are largely lacking, limiting confidence in model generalizability across different clinical settings and ultrasound platforms [3,9,10,11,12,13,14,15,16,17].

AI applications may support standardization across centers and operators, contributing to more consistent bronchoscopic and EBUS practice, while maintaining clinician oversight [3,7,8,17,18]. The authors aimed to develop and evaluate a Vision Transformer-based AI model for automated LN-detection, and characterization (malignant versus benign) during EBUS, using multicenter and histopathologically validated EBUS data.

## 2. Materials and Methods

### 2.1. Study Design and Setting

**Figure 1:**
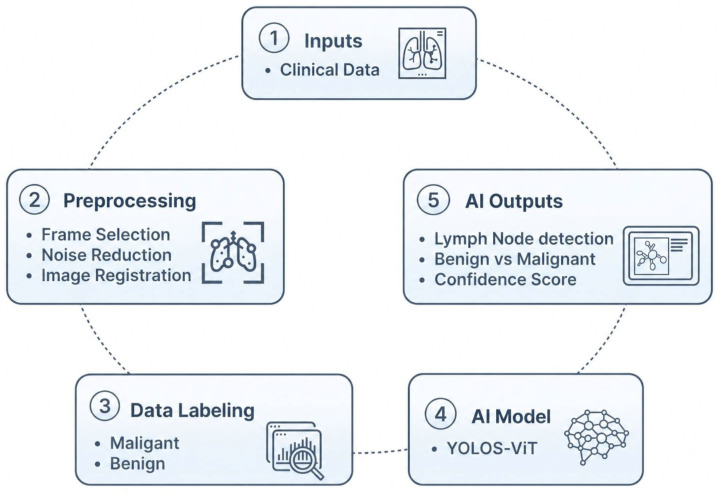
Study flowchart. Abbreviations: AI—artificial intelligence; YOLOS—“You Only Look Once Sequence”; ViT—Vision Transformer-based.

A multicenter retrospective study, using a multi-device image dataset, was conducted, aiming the development and validation of a DL-model for automated-detection and characterization of LNs during EBUS. EBUS images were retrospectively collected from three different reference institutions, in Spain and Brazil: Hospital Universitario Puerta de Hierro (HUPdHM, Madrid, Spain), Hospital das Clínicas da Faculdade de Medicina da Universidade de São Paulo (HCFMUSP, São Paulo, Brazil) and Hospital São Luiz Itaim (HSLI, São Paulo, Brazil). Twenty-seven EBUS videos were collected, from 27 patients that were submitted to EBUS, between 2023 and 2026. The media files from each exam, either full-length videos or still frames, were reviewed. Inclusion and classification of frames were performed by a group of three clinicians with expertise in the interpretation of EBUS. The final decision on frame labeling required the agreement of at least two of the three researchers. The study flowchart is summarized in Figure 1.

This retrospective non-interventional study was approved by the ethics committee of each participating center. The study protocol respects the original and subsequent revisions of the declaration of Helsinki. The output provided by the model had no influence on clinical management of each included patient. Any information susceptible to identify the included patients was omitted, and each patient was assigned a random number in order to guarantee effective data anonymization for researchers involved in the model development. The non-traceability of data and conformity with the general data protection regulation was confirmed by Data Protection team.

### 2.2. Study Population, Image Acquisition and Dataset Curation

Authors developed a DL-system, integrating LN localization and malignant characterization modules. A multi-device dataset from three EBUS platforms was assembled: BF-UCP190F (Olympus™, Tokyo, Japan), BF-UC180F (Olympus™, Tokyo, Japan) and Fujifilm EB-530US (Fujifilm™, Tokyo, Japan). Only complete EBUS exams were included. A complete exam was considered if all stations were described. Inclusion criteria included available EBUS LNs images, histopathologic confirmation of benign versus malignant, and proper image quality. Poor quality or noninterpretable images, duplicated or nonrepresentative frames, and lack of reference standard were considered exclusion criteria. The dataset was divided into training, validation, and testing subsets.

Each frame was processed to remove patient identifiable information (name, operating number, date of procedure). Image annotation was performed by expert pulmonologists on a web-based software (Encord™, Encord Technologies, London, UK, https://app.encord.com). Bounding-box annotations were applied for LN localization. LNs were classified as benign or malignant, according to histopathologic reference standards.

### 2.3. Patients, Lymph Nodes, and Reference Standard

Before interpreting pathology, it is crucial to characterize EBUS results by diagnostic yield. Adequate sample is defined as presence of lymphoid tissue, and sufficient material for cytology/histology. A non-diagnostic sample is considered when it only has blood, bronchial cells without lymphocytes, insufficient cellularity, or when technical sampling failed. EBUS-TBNA cytological results were categorized into four major diagnostic groups: malignant (evidence of malignancy in the sample, confirming N1/N2/N3 disease in LC staging); benign-specific (a defined benign condition was identified, e.g., granulomatous disease, which excluded N1/N2/N3 disease); benign-non-specific/reactive lymphoid tissue (lymphoid tissue present, with no malignant cells or specific pathology; reactive lymphocytes, polyclonal lymphoid population, no granulomas, and no malignant cells; may require repeated EBUS or mediastinoscopy if suspicion remains high); or non-diagnostic, when inadequate material was obtained (may require EBUS, or mediastinoscopy, to be repeated). Benign-specific and benign-non-specific findings were combined and ultimately considered benign findings. Non-diagnostic samples were not considered for establishment of the reference standard for this study.

### 2.4. Model Development

#### Detection and Characterization Module

A real-time box-level deep learning model for LN detection and characterization was developed, with both detection (CADe) and characterization (CADx) features. The model’s output consisted of the identification of the lesion using bounding boxes, along with confidence scores for either class (malignant or benign).

The model was based on YOLOS (You Only Look Once Sequence), a Vision Transformer (ViT)-based object detector with weights pre-trained on ImageNet. To transfer learning to our data, we kept the transformer encoder layers of the model and replaced the detection head with a fully connected classification layer matching the number of target classes (Malignant and Benign), using learned detection query tokens for end-to-end bounding box prediction. To avoid overfitting, a data augmentation pipeline was applied during training, comprising random 90° rotations, horizontal flips, brightness and contrast adjustments, CLAHE, median blur, elastic transformations, and MixUp. The size of each image was set to 640 × 640 pixels. A differential learning rate strategy was adopted, with a backbone learning rate of 5 × 10^−6^ and a detection head learning rate of 5 × 10^−5^, a batch size of 16 with 2-step gradient accumulation (effective batch size of 32), and 36 training epochs. We used PyTorch 2.7.1 (with NVIDIA^®^ CUDA 12.8, NVIDIA Corporation, Santa Clara, CA, USA) and PyTorch Lightning 2.6.1 libraries to prepare the data and run the model. The analyses were performed with a computer equipped with 64 vCPUs, 235 GB of RAM, and an NVIDIA^®^ Tesla T4 graphics processing unit.

### 2.5. Training, Validation, and Testing

#### 2.5.1. Data Partitioning

Dataset was randomly divided into training, validation, and testing subsets, an approximate distribution of 75/15/10 (training/validation/testing), while preserving the class distribution of benign and malignant LNs across subsets through stratified sampling. Model performance was reported for the test set. Data augmentation techniques included random rotations, horizontal flips, scaling, and brightness adjustments to simulate variability encountered in real-world EBUS imaging.

To minimize sampling bias, partitioning was performed at a patient level, ensuring that images or LN samples from the same patient were not distributed across different subsets. This approach allowed prevention of data leakage, and a more reliable assessment of model generalizability. When multiple frames from the same EBUS examination were available, all frames from a given lymph node were assigned to the same dataset partition to avoid information leakage.

#### 2.5.2. Model Performance and Statistical Analysis

For each image, the model calculated the probability for each of the categories (benign vs. malignant). The classification threshold was selected exclusively on the validation set using the Youden Index, which maximizes the trade-off between sensitivity and specificity. A higher probability value translated into greater confidence in the model’s prediction. The category with the highest probability score was outputted as the model’s predicted classification. The output provided by the network was compared to the histopathological gold standard. Given the pilot nature of this study, all results were calculated at an image level. The performance of the CADe component was evaluated with mean average precision at an intersection over union threshold of 0.5 (mAP50). CADx performance was assessed using accuracy, sensitivity, specificity, and area under the receiver operating characteristic curve (AUROC). Statistical analysis was performed using Sci-Kit learn v0.22.2.

## 3. Results

### 3.1. Study Cohort and Dataset Characteristics

A total of 27 patients undergoing EBUS for LN evaluation were included in this study, of which 17 (62.9%) were male. The mean age was 66 ± 16 years. Malignant LNs accounted for 59.3% of all cases. Table 1 summarizes demographical data.

The YOLOS ViT model was developed using a total of 6492, of which 3855 showed malignant LNs and 2637 displayed LNs with no evidence of malignancy. This pool of images was split into training (76.4%, n = 4963), validation (13.5%, n = 876), and testing (10.1%, n = 653) datasets.

### 3.2. Lymph-Node Module Detection Performance

**Figure 2:**
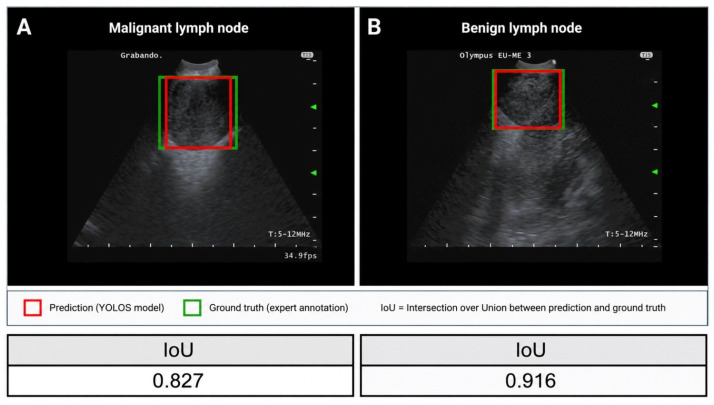
Lymph nodes detected by the YOLOS model with corresponding IoU. (**A**) Malignant lymph node; (**B**) benign lymph node. Abbreviations: IoU—intersection over union; YOLOS—“You Only Look Once Sequence”.

The ViT model evaluated each image and predicted a classification (malignant vs. benign), which was compared with the classification provided by a pulmonologist. Repeated inputs of data to the model resulted in improvements in its accuracy. Figure 2 depicts examples of detected malignant (Figure 2A) and benign (Figure 2B) LNs.

Detection performance was evaluated with mean average precision, at an intersection over union threshold of 0.5 (mAP50). At the detection level, the model showed a mAP50 value of 0.790.

### 3.3. Lymph Node Malignant Characterization Module Performance

**Figure 3:**
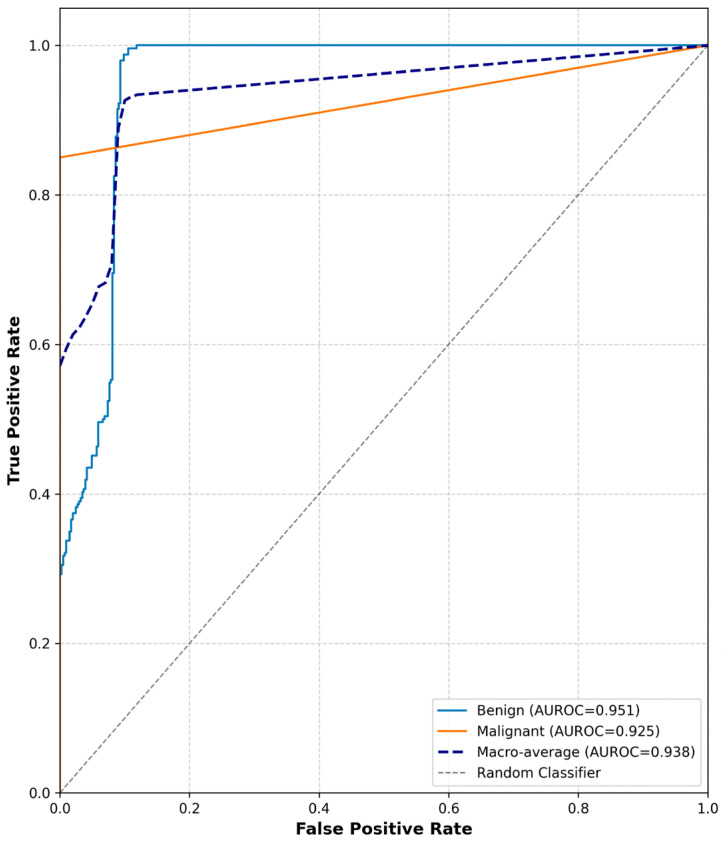
Receiver operating characteristic curve for the characterization of lymph nodes in endobronchial ultrasound images. Abbreviations: AUROC—area under the receiver operating characteristic curve.

Characterization performance was assessed using accuracy, sensitivity, specificity, and AUROC. For malignant characterization, the model achieved an overall accuracy of 90.6% ± 1.3%, sensitivity of 85.4% ± 2.1%, and specificity of 100.0% ± 1.0%. Classification metrics are summarized in Table 2. The model showed an average AUROC of 0.938 (Figure 3).

### 3.4. Error Analysis

**Figure 4:**
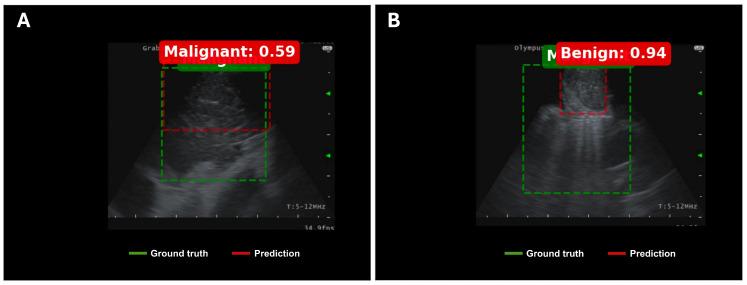
Review of missed detections by the YOLOS model. (**A**) Accurate characterization but missed detection due to intersection over unit under 50%; (**B**) lymph node misclassification, probably due to acoustic shadowing. Abbreviations: YOLOS—“You Only Look Once Sequence.

An error analysis was conducted by reviewing misclassified LNs in the test set, for better understanding of model performance. False-positive predictions most frequently occurred in reactive or inflammatory LNs (which can exhibit heterogeneous echogenicity, irregular margins, or increased vascular patterns that mimic malignant features). In several cases, granulomatous diseases also generated false-positive predictions (image overlap with metastatic involvement). False-negative predictions were mostly observed in small LNs. The smaller the lesion, the higher the possibility of a lower intersection over unit, therefore accounting for failed detections (Figure 4A). Moreover, image-quality limitations such as acoustic shadowing and motion artifacts contributed to several misclassifications (Figure 4B).

## 4. Discussion

Although ML includes algorithms for patter recognition in data, the high visual complexity of endoscopic imaging has shifted most applications toward DL [5,19,20]. Convolutional Neural Networks (CNNs) are the most widely used DL architecture in pulmonary endoscopy, because of their ability to learn hierarchical spatial features directly from image data [21,22].

Regarding EBUS, CNN models have been developed to segment LNs and adjacent structures, facilitating downstream classification and biopsy guidance [3,5,14,21,23]. Transformer-based and hybrid architectures are also emerging, offering improved modeling of global image context, and facilitating multimodal data integration [14,24]. Although most experience remains CT-based, application to endoscopic imaging and CT–endoscopy fusion is expanding as datasets grow.

Pulmonary endoscopy is often a single tool in a more complex multimodal diagnostic environment, including clinical, radiological and endoscopic data. AI systems integrating these complementary information sources are being developed to support lesion characterization, staging and procedural planning [3,9,10,11,12,13,14,15,16,17]. Multimodal approaches have generally shown improved performance compared with single-modality models, mainly in oncological applications [20,22].

This study developed a multicenter, multi-device AI model, based on a ViT architecture, capable of both LN detection and characterization during EBUS. Using histopathology as a reference standard, it has demonstrated feasible promising localization and classification performance, in automated node localization, and malignant prediction. The study has several highlights. Firstly, the model demonstrated high performance levels, with a sensitivity of 85.4%, an overall accuracy of 90.6%, and an AUROC of 0.938. The high sensitivity and negative predictive values are pivotal in the development of computer-assisted reading systems, particularly in the oncological setting, to mitigate the probability of missing lesions, while maintaining adequate specificity. The model moves beyond simple image classification of LN, toward a clinically relevant CADe framework.

An accurate LN assessment is crucial for LC staging and treatment selection. EBUS is not just about image interpretation; it is about selecting and sampling the right LNs during staging. A system that first identifies LNs and then estimates malignancy probability mirrors real procedural workflow, more closely. However, pulmonary endoscopy is performed in visually demanding environments, characterized by limited illumination, motion artifacts, heterogeneous tissue appearance, and restricted fields of view. Procedural completeness and systematic LN assessment are often operator-dependent, which contributes to high variability in diagnostic yield and reproducibility [3,5,9,25]. AI-based quality-monitoring tools are therefore being explored to support more objective, and auditable practice. Moreover, clinical decision-making during bronchoscopy frequently requires the integration of multiple heterogeneous data sources, including real-time endoscopic video, ultrasound imaging, and pre-procedural CT-scan. These challenges can contribute to variability in lesion detection, navigation precision, and procedural decision-making [20,22].

These AI tools have been proposed in EBUS, with the aim of reducing missed lesions, supporting training, and facilitating continuous quality improvement [9]. From identifying a node to characterizing its malignant potential, our work mirrors the clinical workflow of EBUS guided-staging, which aligns AI output with how bronchoscopists examine, target, and sample nodes, without replacing pathology or expert judgment. For mediastinal evaluation, AI-assisted EBUS combined with radiological or metabolic data can improve LN risk stratification beyond conventional sonographic criteria alone [3,7,10,14,15,17]. Such models calculate the probability of malignant LNs, and may support more consistent selection of sampling sites, mainly in LC [7,10,14,15,16,17].

Similarly, Chudobiński et al. developed and retrospectively evaluated the Lymph Node Reporting and Data System (LN-RADS), a standardized ultrasound-based classification system for superficial lymph node assessment [26]. The study included 719 histopathologically validated LNs from 489 patients and demonstrated high diagnostic performance for malignancy prediction, with a sensitivity of 89%, specificity of 85%, and overall accuracy of 87%. LN-RADS stratified lymph nodes into progressively increasing malignancy risk categories and showed substantial interobserver agreement, supporting its reproducibility. Importantly, the system identified a considerable number of malignant lymph nodes measuring less than 10 mm, highlighting the limitations of size-based assessment alone: small malignant lymph nodes may be more difficult to detect and characterize, due to the loss of fine-grained structural information during feature extraction. Authors emphasize the value of integrating multiple sonographic features into standardized risk-stratification frameworks, in order to avoid clinical consequences of false-negative findings in mediastinal staging. These findings suggest that structured LN classification systems may improve diagnostic consistency and facilitate clinical decision-making in oncologic practice. Similarly, AI-assisted EBUS systems may contribute to the standardization of LN characterization by integrating multiple sonographic features beyond size alone, thereby reducing operator dependency and improving reproducibility [26].

These advances in AI have the potential to alter treatment planning in interventional pulmonology. Nevertheless, these predictive and prognostic models require prospective validation and alignment with established staging systems (TNM) before widespread clinical adoption. These tools should be regarded as supportive rather than a replacement to conventional guideline-based algorithms [3,7,27].

AI assistance could support more systematic evaluation of LNs, reducing operator dependence and improving reproducibility. A tool that both localizes suspicious LNs and predicts malignancy could be valuable during real-time procedural decision making. Such assistance could support standardization, training, and reproducibility. Most AI systems designed for pulmonary endoscopy remain at an early stage of clinical maturity. Most studies focus on model development rather than demonstrating clinical impact [3,9,10,11,12,13,14,15,16,17]. While this impact has been explored for gastrointestinal endoscopy, the impact of AI in pulmonary endoscopy technologies is in its exploratory stages. The development of DL models including both CADe and CADx functions is pivotal to provide both visual support and automatic characterization. In the field of EBUS, CADx extends CADe systems’ role by classifying detected findings, and estimating the likelihood of malignancy [22,28].

EBUS interpretation relies on multiple qualitative criteria, being operator-dependent. Zhi et al. developed a ML model to automatically identify informative frames from EBUS strain videos, reducing operator-dependent selection and enabling more standardized downstream interpretation [29]. Ervik et al. demonstrated that CNN-based models can automatically select high-quality convex probe EBUS frames, segment LN boundaries on EBUS imaging, and distinguish LNs from surrounding anatomical structures, with potential to improve transbronchial needle aspiration (TBNA) accuracy and malignancy prediction [14]. In addition, multimodal approaches integrating B-mode imaging, Doppler, and elastography features have been explored for LN classification, with some studies achieving performance comparable to expert assessment. Indeed, AI-enhanced elastography analysis may reduce subjectivity in stiffness pattern assessment [17,30].

Most previous AI studies in thoracic oncology focus on CT, PET, pathology, or navigational bronchoscopy. Far fewer studies have addressed ultrasound-based intraprocedural LN interpretation [5,9,10,11,12,13,14,15,16,17]. Our study adds multicenter, multi-device evidence, and integrates both LN localization and characterization: it extends prior work from isolated classification tasks to a more clinically aligned CADe/CADx framework. This work is among the earliest applications of DL to EBUS with combined detection and classification.

Translation of AI into pulmonary endoscopy depends not only on algorithm performance, but also on dataset quality and dimension. In this sense, the authors must acknowledge that the most prominent limitation of this proof-of-concept study is the small patient and frame sample size. This limitation is motivated by the scarcity of high-quality EBUS data. Despite this limitation, the authors reckon that the goal of demonstrating the feasibility of a CADe/CADx model for detection and characterization of LNs on EBUS images is adequately met. Furthermore, although our study includes exams from several centers, the number of exams from each enrolled center is unbalanced.

Most published datasets do not represent patient diversity (e.g., age, sex, smoking history, comorbid lung disease, disease prevalence), remain single-center, and device-specific, making domain shifts occur across centers, bronchoscopy platforms and ultrasound systems, which poses major challenges to model robustness. Although the present study included a multicenter multidevice dataset acquired from three tertiary referral institutions, the authors must acknowledge that the small sample size hinders large scale representativeness and remains an important limitation. Moreover, the inclusion of consecutive cases led to a slightly unbalanced case distribution, with larger representation of malignant cases. While patient-level data partitioning was used to minimize information leakage and improve the robustness of performance estimates, the possibility of residual overfitting cannot be completely excluded. Proper dataset partitioning is fundamental. Frame-level splits may lead to information leakage, when images from the same patient appear in both training and test sets, inflating performance estimates [14,31]. Patient-, or procedure-level splits are recommended, yet external validation on independent datasets remains uncommon [14,32]. Without such validation, reported performance metrics may not reflect real-world deployment conditions. Therefore, the reported results should be interpreted as a proof-of-concept demonstration of feasibility rather than definitive evidence of clinical effectiveness.

An additional limitation relates to the intrinsic feature extraction mechanisms of DL architectures. Although transformer-based and CNN models are highly effective at learning discriminative image representations, successive feature aggregation and spatial smoothing operations may attenuate fine image details, subtle edge gradients, and micro-textural characteristics. This limitation may be particularly relevant in EBUS imaging, where small LNs, heterogeneous borders, or acoustic shadowing artifacts can contain diagnostically important information. In oncological staging, failure to identify small malignant LNs may lead to disease understaging and potentially impact treatment selection. From a clinical perspective, false-negative classification of small malignant lymph nodes may result in disease understaging and potentially influence treatment selection. Therefore, future research should explore strategies aimed at preserving diagnostically relevant structural information before feature extraction, including morphology-based preprocessing techniques, edge-preserving filters, rank-based connectivity constraints, and other structure-aware image enhancement approaches. Such methods may improve the representation of small LNs and subtle sonographic features, while maintaining the benefits of deep learning-based detection and classification frameworks [3,26].

Future studies should focus on large-scale prospective external validation across diverse populations, operators, and EBUS platforms to establish the generalizability and clinical utility of the proposed system.

An important consideration for future clinical deployment is cross-vendor hardware variability. Differences in ultrasound processors, probes, image acquisition settings, post-processing algorithms, image resolution, contrast, gain, and manufacturer-specific signal processing may affect image appearance and consequently model performance. Although the present study included data acquired from multiple centers and EBUS platforms, larger datasets encompassing a broader range of vendors and acquisition protocols will be required to fully assess model robustness. Future development should deepen this aspect of interoperability, in accordance with the FAIR principles for responsible AI development, and incorporate domain adaptation strategies, vendor-diverse training datasets, calibration procedures, and prospective external validation across different hardware environments to ensure reliable performance and generalizability in real-world clinical practice [3,26,33].

AI is increasingly being applied in pulmonary endoscopy, showing developments in image enhancement, lesion detection, navigation, staging, and procedural support. AI systems have the potential to improve diagnostic consistency, reduce operator variability, and support training in complex bronchoscopy and EBUS procedures [3,9,10,11,12,13,14,15,16,17]. In particular, AI-assisted EBUS LN assessment is feasible, may help in addressing challenges related to variability in mediastinal staging, and shows promise for automated LN localization and characterization. These findings support the further development of real-time decision support systems for minimally invasive thoracic oncology staging. The combination of automated localization and malignant characterization represents an important step toward intelligent procedural support in thoracic oncology [3,5,9,10,11,12,13,14,15,16,17]. Future studies should focus on large-scale prospective external validation across diverse populations, operators, and EBUS platforms to establish the generalizability and clinical utility of the proposed system.

## 5. Conclusions

Researchers developed an artificial intelligence system to help identify and classify lymph nodes during endobronchial ultrasound (EBUS), a procedure commonly used to stage lung cancer [4]. EBUS is effective but depends heavily on the experience of the doctor performing it, which can lead to differences in interpretation. The study used more than 6400 ultrasound images from patients in Spain and Brazil. The AI was trained to locate lymph nodes and determine whether they were benign or cancerous. In testing, the AI was able to detect lymph nodes with good performance and correctly identify malignant nodes in about 91% of cases overall. It detected cancerous nodes with a sensitivity of about 85%, meaning it identified most malignant nodes correctly. These results suggest that AI could help doctors interpret EBUS images more consistently, and potentially improve lung cancer staging, although larger studies are still needed before routine clinical use.

Based on these multicenter study results, the authors reckon that ViT-based AI systems could accurately localize LNs and characterize malignant involvement during EBUS procedures, thereby supporting more reproducible nodal assessment during LC staging, suggesting a potential role for AI-assisted support in LC nodal staging workflows. However, further validation is required before routine use.

## Figures and Tables

**Table 1:** Demographical data.

	Patients (n = 27)
Age, years (SD)	66.0 (15.9)
Sex, n (%)	
Male, n (%)	17 (62.9)
Female, n (%)	10 (37.0)
Collection site, n (%)	
Hospital Universitario Puerta de Hierro Majadahonda	24 (88.9)
Hospital das Clínicas da FMUSP	2 (7.4)
Hospital São Luiz Itaim	1 (3.7)
EBUS Collection Device, n (%)	
Olympus™ BF-UCP190F	24 (88.9)
Fujifilm™ EB-530US	2 (7.4)
Olympus™ BF-UC180F	1 (3.7)
Histopathological Diagnosis, n (%)	
Malignant	16 (59.3)
Benign	11 (40.7)

Abbreviations: FMUSP—Faculdade de Medicina da Universidade de São Paulo; EBUS—Endobronchial Ultrasound; SD—standard deviation.

**Table 2:** Performance of a CADe system for characterization (CADx component) of lymph nodes in endobronchial ultrasound.

	Performance
Sensitivity, % (95% CI)	85.4 (85.3–85.5)
Specificity, % (95% CI)	100.0 (99.9–100.0)
PPV, % (95% CI)	100.0 (99.9–100.0)
NPV, % (95% CI)	79.8 (79.6–80.0)
Accuracy, % (95% CI)	90.6 (90.5–90.7)
F1-Score, % (95% CI)	92.0 (91.9–92.1)

Abbreviations: 95% CI: 95% confidence interval; NPV—negative predictive value; PPV—positive predictive value.

## Data Availability

The data presented in this study are available on request from the corresponding author, upon reasonable request.

## Abbreviations

The following abbreviations are used in this manuscript:

AI	Artificial Intelligence
AUROC	Area Under the Receiver Operating Characteristic Curve
CADe	Computer-Aided Detection
CADx	Computer-Aided Diagnosis
CC BY	Creative Commons Attribution
CLAHE	Contrast Limited Adaptive Histogram Equalization
CNN	Convolutional Neural Network
CT	Computed Tomography
CUDA	Compute Unified Device Architecture
DL	Deep Learning
EBUS	Endobronchial Ultrasound
FAIR	Findable, Accessible, Interoperable and Reusable
FMUSP	Faculdade de Medicina da Universidade de São Paulo
HCFMUSP	Hospital das Clínicas da Faculdade de Medicina da Universidade de São Paulo
HSLI	Hospital São Luiz Itaim
HUPdHM	Hospital Universitario Puerta de Hierro Majadahonda
ImageNet	Large-scale image database used for pretraining
IoU	Intersection over Union
LC	Lung Cancer
LN(s)	Lymph Node(s)
LN-RADS	Lymph Node Reporting and Data System
mAP50	Mean Average Precision at IoU threshold = 0.5
ML	Machine Learning
NLP	Natural Language Processing
NPV	Negative Predictive Value
PET	Positron Emission Tomography
PPV	Positive Predictive Value
RAM	Random Access Memory
ROC	Receiver Operating Characteristic
SD	Standard Deviation
TBNA	Transbronchial Needle Aspiration
TNM	Tumor–Node–Metastasis
vCPU	Virtual Central Processing Unit
ViT	Vision Transformer
YOLOS	You Only Look One Sequence
